# Peptides for trans‐blood–brain barrier delivery

**DOI:** 10.1002/jlcr.4023

**Published:** 2023-04-20

**Authors:** Reuben Blades, Lars M. Ittner, Ole Tietz

**Affiliations:** ^1^ Dementia Research Centre, Macquarie Medical School, Faculty of Medicine, Health and Human Sciences Macquarie University Sydney New South Wales Australia

**Keywords:** blood–brain barrier, cell penetrating peptides, peptides, radiopharmaceuticals

## Abstract

Trans‐blood–brain barrier (BBB) delivery of therapeutic and diagnostic agents is a major challenge in the development of central nervous system (CNS) targeted radiopharmaceuticals. This review is an introduction to the use of peptides as delivery agents to transport cargos into the CNS. The most widely used BBB‐penetrating peptides are reviewed here, with a particular emphasis on the broad range of cargos delivered into the CNS using these. Cell‐penetrating peptides (CPPs) have been deployed as trans‐BBB delivery agents for some time; new developments in the CPP field offer exciting opportunities for the design of next generation trans‐BBB complexes. Many of the peptides highlighted here are ready to be combined with diagnostic and therapeutic radiopharmaceuticals to develop highly effective CNS‐targeted agents.

## Graphical abstract

Trans‐blood–brain barrier (BBB) delivery of therapeutic and diagnostic agents is a major challenge in the development of central nervous system (CNS) targeted radiopharmaceuticals. This review is an introduction to the use of peptides as delivery agents to transport cargos into the CNS. Peptides highlighted here are ready to be combined with diagnostic and therapeutic radiopharmaceuticals to develop the next generation of CNS‐targeted agents.

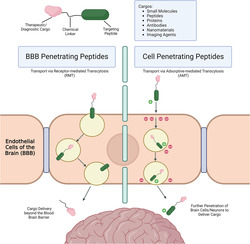

## 1 INTRODUCTION

The blood–brain barrier (BBB) is a major obstacle to the delivery of diagnostic and therapeutic agents to the central nervous system (CNS) and constitutes a major obstacle in the treatment and diagnosis of CNS diseases and conditions.^1^ The BBB is a vast multicellular dynamic interface that controls the passage of specific nutrients from the vasculature to the extracellular fluid of the CNS. It restricts the passage of xenobiotic molecules including neurotoxic agents and protects the brain from adverse effects. As such, the BBB prevents drugs, particularly large molecules such as biopharmaceuticals from entering the CNS.^2^ Levels of exogenous antibodies in the CNS, for example, have been found to correspond to only 0.01–0.1% of those found in plasma.^3^

### 1.1 Peptide—Cargo conjugates

Peptides are a promising class of molecules for the non‐invasive delivery of therapeutic and diagnostic agents into the brain,^4^, ^5^ with a higher risk/reward ratio compared with invasive procedures, which have several limitations. The use of ultrasound for BBB transient disruption (breakage of tight junctions of endothelial cells) allows for molecules to travel into cerebral tissue but does compromise the BBB leading to adverse complications and injury to the CNS. Other procedures involve direct injection of therapeutic agents into the brain, bypassing the need to penetrate the BBB, but these do require sophisticated surgery and are unfavourable for the patient.^6^ Peptides are chains of between 2 and 50 amino acids that have been investigated extensively due to their low cost and ease of synthesis. Peptide sequences are easy to incorporate or conjugate to molecules of interest to improve BBB‐penetrating properties of the cargo. Peptide–drug conjugates (PDCs), consisting of BBB‐penetrating peptide, chemical linker, and payload/cargo, offer enhanced treatment options for CNS‐based diseases.^7^, ^8^

### 1.2 Trans‐BBB delivery pathways

**FIGURE 1:**
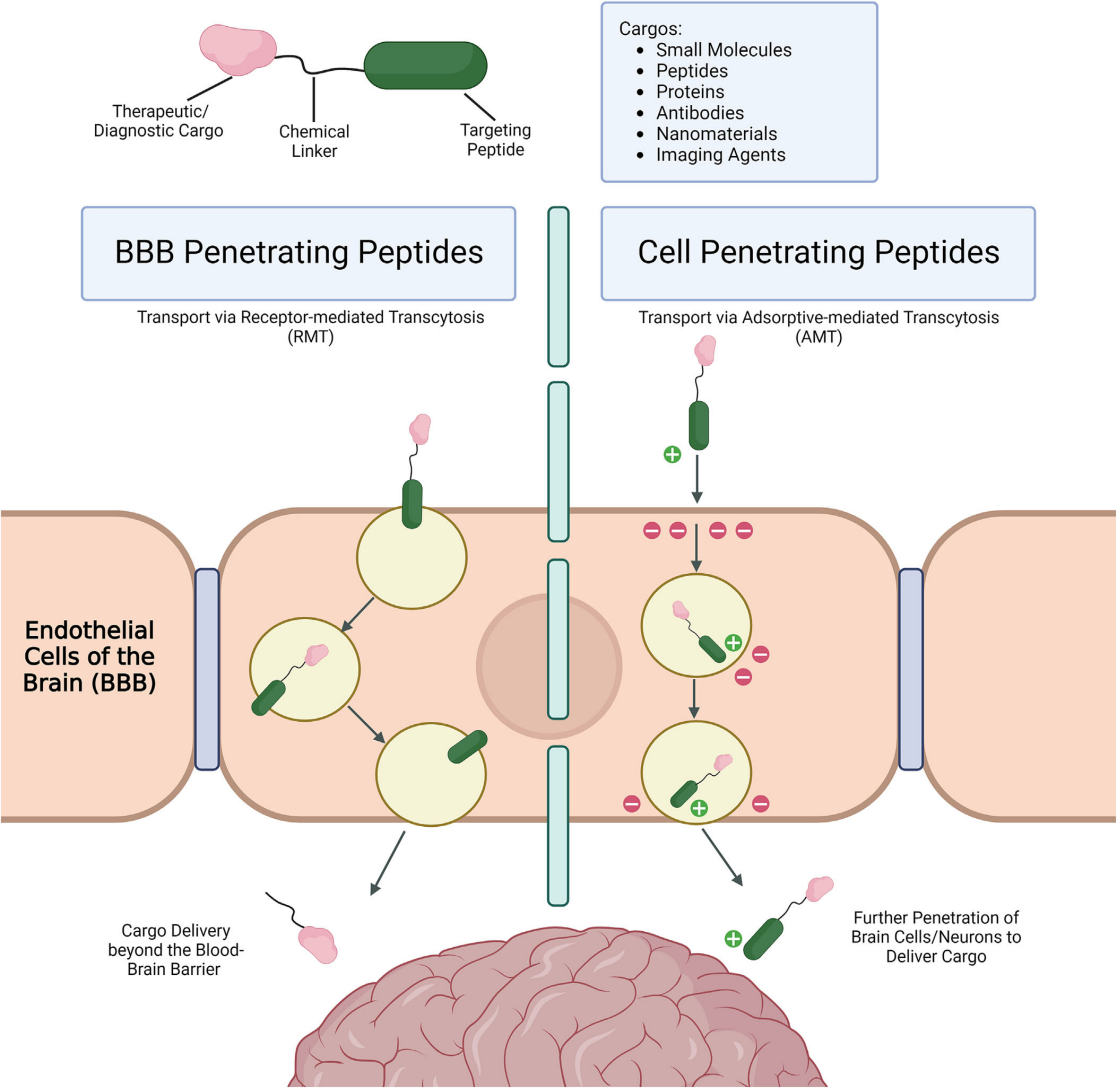
Overview of trans‐BBB delivery of cargos using BBB‐penetrating and cell‐penetrating peptides. BBB, blood–brain barrier.

There are several transport mechanisms that allow molecules and solutes to cross the BBB, these have been reviewed in detail elsewhere.^2^ Advances in the field, particularly aided by cryo‐electron microscopy to resolve the structure of BBB membrane bound receptors, continue to open new avenues of investigation for new trans‐BBB peptides.^9^ An appreciation of these mechanism and how they interact with peptides is beneficial for appropriate peptide–cargo selection (see Figure 1).

Peptides that traverse the BBB are broadly classified into two groups: Peptides with strong affinity towards a specific receptor undergo receptor‐mediated transcytosis (RMT) and are generally referred to as BBB‐penetrating peptides.^7^ Peptides that do not bind to a specific receptor to penetrate the BBB, but instead exhibit broad cell‐penetrating properties, are known as cell‐penetrating peptides (CPPs).^10^ Cationic CPPs can directly penetrate the BBB through adsorptive‐mediated transcytosis pathways, due to electrostatic interactions between the cationic peptide and anionic microdomains of the BBB.^2^ RMT is often exploited in the field of neurotherapeutics, due to the upregulation of specific receptors caused by some CNS diseases. A ligand (drug) with high affinity to the upregulated receptor can be used to bind to the receptor, when receptor‐mediated endocytosis can occur (ligand is engulfed by the cell).^2^ This is followed by the movement of the ligand–receptor complex until the ligand can be deposited on the abluminal side of the BBB.^11^ Adsorptive‐mediated transcytosis is a nonspecific transport mechanism and is instigated by electrostatic interactions between positively charged molecules and the negatively charged membrane of endothelial cells of the BBB.^7^ Although BBB‐penetrating peptides are highly specific for BBB receptors and generally do not interact with other cell types or tissues, CPPs are prone to interact broadly, with many cell types and tissues. One of the advantages of using CPPs is their ability to deliver into neurons in addition to trans‐BBB delivery, thus enabling the targeting of intracellular proteins with a variety of cargoes.^12^ Both BBB‐ and CPP‐type peptides can be used to develop new generations of radiopharmaceuticals targeting the CNS.

Here, we provide a brief introduction to the use of peptides as trans‐BBB delivery agents. We briefly discuss some widely used peptides for trans‐BBB delivery with a particular emphasis on studies that investigated CPPs for the delivery of cargos into the CNS and how these could be applied to the development of radiopharmaceuticals.

## 2 MAIN TEXT

### 2.1 CNS‐targeted radiopharmaceuticals

Radiopharmaceuticals find broad application in the CNS, in particular for the assessment and diagnosis of neurological and neurodegenerative conditions,^13^, ^14^ as well as the diagnosis and treatment of primary brain cancers and metastatic lesion in the brain.^15^, ^16^, ^17^ The majority of radiopharmaceuticals, particularly small molecules, used in the CNS, possesses intrinsic BBB‐penetrating properties. The most prominent example of this type of radiopharmaceutical is ^18^F‐FDG, which is used to assess glucose metabolism and is used for diagnostic purposes in both neurology and neuro‐oncology. Furthermore, this category includes positron emission tomography (PET) tracers for amyloid deposits (^11^C‐PiB), tau deposits (^18^F‐AV‐1451), neuroinflammation (TSPO‐PET–^11^C‐PK11195), and synaptic density (SV2A‐PET–^11^C‐UCB‐J), which are used in the diagnosis of neurodegenerative disease,^13^ as well as amino acid PET tracers (^11^C‐MET, ^18^F‐FDOPA, and ^18^F‐FET) and somatostatin receptor (SSTR) ligands (^68^Ga‐DOTATATE and ^68^Ga‐DOTATOC), which are used in neuro‐oncology.^15^

Larger molecules, including nanomaterials, antibodies, proteins, and some peptides, generally do not have intrinsic BBB‐penetrating properties. This deprives the field of CNS‐targeted radiopharmaceuticals of powerful targeting molecules for the development of novel, highly specific diagnostic and therapeutic agents. A case in point is the field of neurodegenerative disease diagnosis. Proteopathies such as Alzheimer's disease and Parkinson's disease are characterised by aggregated forms of a protein or proteins that are high post‐translationally modified and adopt distinct fibrillary structures.^18^, ^19^, ^20^ Small‐molecule radioligands are unlikely to be specific enough to distinguish these proteoforms to discriminate different isoforms of aggregate protein. The last decade has witnessed the development of effective and specific antibodies for different types of aggregate protein, which could serve as templates for the development of radiopharmaceuticals for the specific diagnosis of neurodegenerative diseases.^21^ The development of CNS‐targeted antibodies has focused on the use of the transferrin receptor (TfR) as an RMT entry point.^22^ These efforts have led to the development of ^124^I‐labelled bispecific antibodies that bind the TfR for efficient entry into the CNS and amyloid‐β (Aβ) once in the brain.^23^, ^24^, ^25^ Recent efforts have focused on reducing the size of agents to achieve improved clearance from the blood pool.^26^ Preclinical studies demonstrated that bispecific antibody [^124^I]RmAb158‐scfv8D3 readily detected changes in Aβ levels at an age when ^11^C‐PiB hardly detected any Aβ.^27^ These studies demonstrate the utility of CNS‐targeted antibody radiopharmaceuticals; the reduction in size and improvement in pharmacokinetic properties offered by peptide conjugates could further enhance the performance of these agents.

The delivery of nanomaterials into the brain is an active area of research and promises to provide an effective theragnostic platform for diagnosis and therapy of brain tumours.^28^ The development of ^64^Cu‐labelled micelle as a highly stable, long‐circulating, surface‐modified PET imaging agent is a notable development in this field.^29^ Further modification with peptides to enhance trans‐BBB delivery is a promising avenue of further research.

A notable exception to the rule that only small molecules possess intrinsic ability to enter the CNS are the SSTR ligands. Somatostatin is a cyclic peptide produced by the hypothalamus and has high affinity to SSTRs (SSTR‐1 to SSTR‐5) which are overexpressed in many forms of cancer, especially those of neuroendocrine origin.^30^, ^31^ For example, SSTR‐2 is highly expressed in gliomas and pituitary brain tumours. Somatostatin can deliver radiopharmaceuticals for both therapeutic and diagnostic purposes. The most popular radiolabelled somatostatin analogues for therapeutic purposes in clinical practice are ^90^Y‐DOTATOC and ^177^Lu‐DOTATATE.^30^ In addition to prolific use outside the CNS, somatostatin radiopharmaceuticals are effective theranostic agents in the CNS.^32^, ^33^, ^34^

Analogues of the naturally occurring, 11 amino acid neuropeptide, substance P, constitute another class of theranostic agents with intrinsic CNS‐targeting properties.^35^ DOTA‐modified substance P was radiolabelled with alpha emitting ^213^Bi and ^225^Ac to deliver short range alpha radiation to gliomas with good efficacy reported in preclinical and clinical studies. A clinical trial with nine secondary glioblastoma multiforme patients reported median overall survival of 16.4 months after the start of ^213^Bi‐Substance P treatment, compared with literature values of 7.8 months following partial surgical resection only, 10 months with radiotherapy only, and 2 month without radiotherapy. Although control groups were not included in this clinical trial, the results suggest utility for substance P alpha therapy as a safe and well‐tolerated therapeutic option in glioblastoma multiforme. Substance P, radiolabelled with ^68^Ga, was furthermore utilised for diagnostic purposes for the use in PET imaging (^68^Ga‐DOTA‐SP), demonstrating the utility of substance P as a theranostic agent.^36^

Another notable addition to the field of CNS‐targeted small peptides is the development of Caspase 3 cleavable ^68^Ga‐DOTA complexes that incorporate a Tat sequence for CNS and intraneuronal delivery.^37^, ^38^ These agents have shown preclinical promise as apoptosis imaging agent in transgenic mouse models of Alzheimer's Disease, where they showed significantly higher accumulation in transgenic versus wild‐type animals consistent with histology and disease burden.^37^ Further, higher uptake was also reported in a preclinical middle cerebral artery occlusion model of stroke in comparison with control animals,^38^ highlighting the promise of these agents as apoptosis imaging agents in CNS.

Peptides are well suited to constitute part of a radiopharmaceutical, due to relatively low molecular weight and favourable pharmacokinetic properties including rapid diffusion into target tissue and clearance from the blood resulting in high contrast. Peptides are cheap and easy to synthesise; in addition, most peptides are capable of enduring harsh radiolabelling conditions.^39^ Consequently, peptides have been used widely as radiopharmaceuticals^40^, ^41^, ^42^, ^43^ and as agents to modulate the pharmacokinetic properties radiopharmaceuticals.^44^, ^45^, ^46^, ^47^, ^48^ The use of peptides in the development of CNS‐target radiopharmaceuticals is comparatively limited by comparison. In the following sections, we provide a brief overview of the use of peptides in nonradioactive therapeutic and diagnostic agents and discuss how these might be utilised in the development of novel CNS‐target radiopharmaceuticals.

### 2.2 Peptides for delivery of cargos across the BBB

Research over the past two decades has identified an arsenal of peptides capable of penetrating through the BBB and combined these with a variety of molecular cargos. Table 1 summarises some of the most prominent peptides which demonstrated the ability to transport cargos across the BBB.

**TABLE 1:** BBB‐penetrating peptides and cell‐penetrating peptides.

Peptide	Peptide type	Sequence	Origin	Transport mechanism	Cargos
Transferrin	BBB penetrating	THRPPMWSPVWP (THR), HAIYPRH (HAI), CRTIGPSVC (CRT), and others	Endogenous protein analogues	RMT (TfR)	Antibodies,^58^ peptides,^49^ and nanomaterials^54^, ^55^, ^56^, ^57^
Angiopep‐2	BBB penetrating	TFFYGGSRGKRNNFKTEEY	Neurotropic endogenous protein	RMT (LRP‐1)	Small drugs,^63^, ^64^ proteins,^65^ and nanoparticles^66^
RGD	BBB penetrating	RGD	Extracellular matrix protein fibronectin	RMT (integrin α_v_β_3_)	Polymers,^69^ radiolabelled peptides,^67^ and micelles^70^
Glutathione	BBB penetrating	γ‐L‐Glutamyl‐L‐cysteinylglycine	Endogenous peptide	GSH transporters	Nanoparticles^73^, ^74^
RVG29	BBB penetrating	YTIWMPENPRPGTPCDIFTNSRGKRASNG	Neurotropic exogenous protein	RMT (nAchR)	Nanoparticles^81^ and DNA/RNA^79^
PepH3	CPP	AGILKRW	Dengue virus capsid protein	Passive diffusion/AMT	Proteins^89^ and nanoparticles^90^
TAT_(47‐57)_	CPP	YGRKKRRQRRR	HIV‐1	AMT	Small drugs,^98^ peptides,^92^ and nanoparticles^95^, ^96^, ^97^
Penetratin	CPP	RQIKIWFQNRRMKWKKGG	Exogenous protein	AMT	Small drugs,^103^ proteins,^104^ and nanoparticles^102^
SynB1	CPP	RGGRLSYSRRRFSTSTGRA	Protegrin I (toxin)	AMT	Small drugs^106^, ^107^

Abbreviations: AMT, absorption‐mediated transcytosis; BBB, blood–brain barrier; CPP, cell‐penetrating peptide; GSH, glutathione; HIV‐1, human immunodeficiency virus 1; LRP‐1, lipoprotein receptor‐related protein 1; nAchR, nicotinic acetylcholine receptor; RGD, arginine–glycine–aspartate; RMT, receptor mediated transcytosis; TfR, transferrin receptor.

Peptides derived from the serum protein transferrin, which binds the TfR, are widely used as BBB‐penetrating peptides.^49^ The TfR is highly expressed in the brain capillary endothelial cells and serves as an attractive target to facilitate transcytosis across the BBB.^50^ THR (THRPPMWSPVWP), HAI (HAIYPRH), and CRT (CRTIGPSVC) peptides have been shown to be highly efficient at binding to TfR and initiating transcytosis in preclinical studies.^51^, ^52^ These peptides have been used to transport liposomes,^53^ nanoparticles,^54^, ^55^ and adeno‐associated viruses^56^ into the CNS. TfR‐mediated transport has been extensively researched and identified as a promising BBB target for drug delivery into the CNS.^57^, ^58^, ^59^

Angiopep‐2 is a BBB‐penetrating peptide with a high affinity to low‐density lipoprotein receptor‐related proteins (LRPs). LRP‐1 and LRP‐2 are large endocytic receptors involved in lipoprotein metabolism.^60^ When tested in an in vitro assay (BBB bovine brain capillary endothelial cell), angiopep‐2 demonstrated over 50 times more transcytosis capacity than transferrin.^61^ Near‐infrared fluorescent imaging of angiopep‐2 using a Cy5.5 fluorescent dye followed by fluorescence analysis shows that angiopep‐2 is rapidly transported into the brain parenchyma.^62^ Angiopep‐2 has also proven to be versatile in the type of cargo that it can transport as a PDC. Initially, angiopep‐2 was designed for transporting small drug molecules such as paclitaxel (PTX) and doxorubicin.^63^, ^64^ Subsequently, angiopep‐2 was shown to transport antibodies and carbon nanotube through the BBB.^65^, ^66^ Angiopep‐2 demonstrates remarkable versatility as a BBB‐penetrating peptide for the delivery of different sized cargos for the treatment of CNS diseases.

Arginine–glycine–aspartate or RGD is a tri‐amino acid sequence that contains a principal binding domain to α_v_β_3_ integrin and is widely utilised to carry drugs to the brain due to the overexpression of α_v_β_3_ integrin in diseases such as brain tumours.^67^ The RGD peptide can be modified to deliver polymers, micelles, liposomes, and radiolabels for therapeutic or diagnostic purposes.^68^, ^69^, ^70^, ^71^, ^72^

Glutathione (GSH) is a BBB‐penetrating peptide that has antioxidant properties and is popular for the delivery of therapeutic nanoparticles into the brain through interaction with GSH transporters.^73^, ^74^ The success of GSH as a BBB‐penetrating peptide is exemplified by the development of G‐Technology®, which uses GSH‐targeted PEGylated liposomes.^73^ Recently, GSH‐targeted PEGylated liposomes have shown to efficiently deliver the anticancer drug doxorubicin into a mouse brain tumour.^75^ G‐Technology® is another promising advancement for the delivery of therapeutic and diagnostic agents into the brain.

Rabies virus glycoprotein (RVG29) contains a 29‐amino acid sequence which binds competitively to nicotinic acetylcholine receptors.^76^, ^77^ RVG29 penetrates the brain parenchyma and can transport drugs, genes, and nucleic acids through the BBB and into the brain via nicotinic acetylcholine receptors.^78^, ^79^ RVG29 is an attractive peptide for the delivery of therapeutics to treat a range of diseases, from Parkinson's to malignant brain tumours such as gliomas.^80^ An example of RVG29's therapeutic potential is the transportation of siRNA to attenuate the long‐term effects of traumatic brain injury.^79^ In a more recent study, RVG29 was used to deliver macrophage membrane‐coated solid‐lipid nanoparticles to target neuronal mitochondria.^81^ Neuronal mitochondria dysfunction is often observed in sporadic Alzheimer's disease and is caused by excessive reactive oxygen species.^81^ RVG29 is used to deliver functional antioxidants into the brain and could be used to delay the progression of Alzheimer's disease. This is a further example of the utility of peptides as delivery vectors across the BBB.

### 2.3 CPPs for delivery into the brain

CPPs are a broad and diverse subset of peptides, consisting of short amphipathic or cationic peptide sequences that can traverse the BBB and translocate cell membranes without compromising their integrity.^82^, ^83^, ^84^ Over 2000 naturally occurring and synthetic peptide sequences have been described as having cell‐penetrating properties to date.^85^ The emergence of CPPs into the field of neuroscience is a result of continued research into the naturally occurring proteins, most prominently the transactivating transcription factor Tat of the human immunodeficiency virus (HIV‐1).^2^ CPPs are used in combination with cargo molecules, such as proteins, nucleic acids, or nanoparticles, which would not be able to penetrate the BBB effectively. CPPs are often advantageous over other delivery mechanisms due to their high permeability, larger cargo capacity, and their ability to infiltrate a wide variety of cell types.^86^ A specific example of this is PepH3 derived from the Dengue virus type 2 capsid protein.^87^, ^88^ Dengue virus type 2 capsid protein‐derived peptides have the ability to deliver cargos, including antibodies,^89^ into the cell.^90^ In addition, in vivo and in vitro *assays* have shown that PepH3 is able to penetrate the BBB via adsorptive‐mediated transcytosis as well as return to the circulatory system for excretion.^87^ Therefore, PepH3 shows promising CPP capability and could be used to ferry drug molecules through the BBB to the brain, particularly for the development of diagnostic agents, where the extraction of unbound molecules from the brain is essential to generate contrast.

Another example of a CPP that has advanced the field of neurotherapeutics is the Tat peptide, derived from the HIV virus.^91^ Tat has been used in one of the most prominent clinical trials of a peptide to treat stroke described in the ESCAPE‐NA‐1 trial conducted by Hill et al.^92^ In this trial, nerinetide (Tat‐NR2B9C) was under investigation for use as an intraneuronal therapy to prevent ischaemic brain damage. Nerinetide is composed of NR2B9c, which is an eicosapeptide (oligopeptide composed of 20 amino acid monomers) which inhibits the postsynaptic density‐95 protein, disturbing protein–protein interactions,^92^, ^93^ and Tat which facilitates transport across the BBB and into the neuron. This disruption between proteins prevents neurotoxic signalling in events of acute ischaemic stroke.^93^ After extensive validation of nerinetide in primate models, the drug was assessed for efficacy in reducing the number of ischaemic strokes in human patients during surgery for endovascular repair of intracranial aneurysms.^92^ The ESCAPE‐NA‐1 trial ultimately failed to produce new and effective drugs to combat acute ischaemic strokes, but this was later shown to be due to the proteolytic cleavage of Tat, especially when co‐administered with the drug alteplase.^94^ The ESCAPE‐NA‐1 trial demonstrated that Tat is a highly effective delivery agent for peptide therapeutics and diagnostics into the CNS and the neuron, especially if proteolytically stable Tat analogues, such as cyclic Tat or D‐Tat, are used. Tat has further been used to transport nanoparticles^95^, ^96^, ^97^ and small molecule drugs^98^ into the CNS. The unique design of nerinetide and other Tat conjugates sets the groundwork for future work in CNS therapeutics, and further studies are needed to assess the effectiveness of Tat as a CPP for use in the CNS.^99^

Penetratin, derived from the Drosophila Antennapedia protein, is one of the archetypal CPPs. Much like Tat, penetratin can traverse the BBB via adsorptive‐mediated transcytosis and is used either alongside another peptide or by itself.^100^, ^101^ Penetratin can deliver a wide variety of cargos, such as small drug molecules, imaging agents, oligonucleotides, and nanoparticles.^102^, ^103^, ^104^ In a recent example, penetratin was used in combination with transferrin to deliver liposomes loaded with a plasmid encoding the Apolipoprotein E2 gene, for improved brain delivery in a preclinical model.^105^ Apolipoprotein E2 gene contributes to neuronal function and uptake in the brain could be used to treat Alzheimer's disease. The study concluded that a dual approach of transferrin–penetratin was effective for enhanced brain targeting and therapeutic gene delivery.^105^

SynB1 is an 18‐residue CPP derived from the antimicrobial peptide protegrin 1.^106^ Small drugs such as doxorubicin and benzylpenicillin have been proved to have an increase uptake in the brain when paired with SynB1 as a delivery vector.^107^ For example, the accumulation of the antibiotic benzylpenicillin in the brain when coupled with SynB1 increases by a factor of 7, compared with free ^14^C‐labelled benzylpenicillin. Further studies showed that the vectorised drug showed no loss of integrity and was distributed around all assessed areas of the brain.^108^, ^109^, ^110^

### 2.4 New CPP approaches

There have been a number of seminal developments in the field of CPPs over the past 5 years that have brought the possibility of effective intracellular diagnostics and therapeutics closer to clinical reality. These novel agents have not been investigated for their ability to traverse the BBB or their potential to compliment neurotherapeutics to date. However, the success of agents like nerinetide and the crucial role that the CPP Tat plays in delivering cargos into the CNS and the neuron suggest that these second generation CPPs are likely to yield highly effective trans‐BBB delivery agents that merit further investigation and inclusion on future efforts to design CNS radiopharmaceuticals.

Multimerisation and clustering strategies that serve to connect multiple copies of CPP with their intended cargo are characteristic attributes of second generation CPPs. A recent study successfully demonstrated the uptake of antibodies into the cytosol and nucleus of live cells by co‐delivering them with a trimer of cyclic Tat peptides resulting in delivery efficacy more than 10‐fold better than that of single, linear Tat peptide as used in nerinetide.^111^ Another study showed the efficient cytosolic delivery of the antibodies by delivering it using a trimeric version of natural toxin‐derived CPP L17E.^112^ As demonstrated with Tat, trimerisation of the L17E lead to a marked increase in intracellular uptake compared with monomers. Tetramerisation of CPPs has also been shown to improve the cytosolic delivery of monoclonal antibodies into cells.^113^ More general approaches of multimerisation have also shown to be highly efficient. Delivery of CPP–antibody conjugates when co‐delivered with thiol‐reactive CPP additives was used to accelerate the formation of disulphide bridges on the cell surface and deliver therapeutics into cell.^114^ Finally, clustering of peptides into coacervates has been shown to be viable delivery option for a range of cargos into cytosol. Therapeutic‐loaded peptide coacervates were shown bypass endocytic entrapment, successfully delivering anti‐cancer peptides and other biomacromolecules into the cytosol.^115^

Whether these approaches can improve peptide‐assisted trans‐BBB delivery remains to be established, but these studies provide a useful blueprint for the design of next generation CNS delivery systems with increased efficacy and safety profiles.

### 2.5 Range of cargos delivered into the brain

Peptides that can penetrate the BBB facilitate CNS delivery and generally do not have additional diagnostic or therapeutic function; they are therefore usually linked to cargos that mediate the desired function. In principle, any cargo can be delivered using these peptides including small drug molecules, peptides, proteins, antibodies, nanoparticles, and imaging agents.^116^ However, the type of cargo that can be transported varies from peptide to peptide and is generally linked to the mechanism of uptake that a specific peptide is able to access (discussed below). The following will provide a brief overview of PDCs to illustrate the use of appropriate peptide for a specific cargo.

Small molecule drugs that do not cross the BBB by themselves have been linked to peptides to facilitate CNS delivery. PTX is a microtubule inhibitor anticancer drug, typically used to treat ovarian, breast, and nonsmall cell lung cancers, as well as acquired immunodeficiency syndrome (AIDS)‐related Kaposi's sarcoma in the clinic.^117^ Although an effective drug, PTX is highly hydrophobic and requires a delivery vector to traverse the BBB so it can bind to tumour tissue. A recent example used a combination of Tat and Angiopep‐2 as dual‐vectors to deliver PTX to the brain to treat gliomas.^98^ Another anticancer drug that has gained clinical approval is doxorubicin. Doxorubicin is used in the treatment of bladder and breast cancer, Kaposi's sarcoma, and acute lymphocytic leukaemia and is widely implemented with BBB‐penetrating peptides as PDCs.^118^ CPPs such as SynB1 and penetratin have both been used alongside doxorubicin to treat primary brain tumours such as glioblastoma.^103^, ^119^

In addition to small molecule drugs, peptides and antibodies have also been delivered into the brain for therapeutic purposes. Neurotensin is a naturally occurring peptide in the CNS and regulates nociceptive transmission (pain in the nervous system caused by inflammation or disease). Neurotensin provides an analgesic effect when applied directly to the brain and has been linked to the BBB‐penetrating peptide angiopep‐2 as a delivery vector.^120^ Anti‐HER2 monoclonal antibodies are effective treatment strategies for reducing the size of breast cancers and are under investigation for the treatment of breast cancer‐related brain metastases.^62^ A recent study investigated whether transferrin attached to the therapeutic antibody trastuzumab and mucic acid polymer (conjugate of the anticancer drug camptothecin) could be used to inhibit tumour growth.^121^ The combination of antibody and drug delivered by the BBB‐penetrating peptide exhibited a considerable antitumour response for brain metastases in a mouse model.^121^

## 3 PERSPECTIVE

There is an urgent need for the development of new and effective strategies to deliver therapeutic and diagnostic agents into the CNS. The failure rate of CNS‐targeted therapies in clinical trials is about twice that of non‐CNS‐targeted drugs^122^; for some neurodegenerative disease, such as Alzheimer's disease, the failure rate is close to 100%.^123^ Although the reasons for these failures are manifold and undeniably complex, drug delivery across the BBB is a major contributor to failure at preclinical and clinical stages, as recently highlighted in field of immunotherapy for neurodegenerative diseases.^124^

Delivery across the BBB is a particularly tall obstacle for the development of CNS‐targeted radiopharmaceuticals. Internal radionuclide therapies are highly effective at treating metastatic cancer lesion but are less effective against brain metastasis which are a major challenge in cancer therapy.^124^ The progression of neurodegenerative diseases remains poorly understood due to the unavailability of tissue samples at different disease stages. PET is one of few technologies that can provide functional and mechanistic insight into the progression of neurodegenerative diseases. However, the development of the new PET tracers, particularly against specific conformations of misfolding proteins such as β‐amyloid, tau, and α‐synuclein, struggles with access to the CNS.^13^

A specific challenge in the development of CNS radiopharmaceuticals is the need to generate contrast and the associated requirement for CNS efflux as well as influx. PET radiopharmaceuticals are only useful if they can generate high signal‐to‐noise ratios in a time frame that is compatible with the half‐life of their radionuclide. PET tracers that are not target bound but give rise to signal in the brain because of slow efflux kinetics across the BBB will obscure target specific signal. This requirement also applies to therapeutic radiopharmaceuticals, particularly those with radionuclides that emit long‐range ionising radiation, such as gamma and beta emitters. Slow efflux of nontarget bound therapeutics would lead to irradiation of healthy brain tissue and detrimental side effects. This problem is somewhat less severe with shorter range radionuclides, such as alpha and Auger emitters, but can still cause unwanted effects on healthy tissues. Peptides with BBB influx as well as efflux function, such as PepH3, might therefore be most suitable for the development of CNS radiopharmaceuticals. Furthermore, CPPs that have transcytosis function without binding to specific receptors might be particularly suitable for the development of agents that readily transit to BBB bidirectionally.

There are number of new technologies emerging in the field of peptide‐mediated drug delivery that merit further investigation for CNS delivery. Multimeric and clustering CPP approaches have been shown to be highly effective at delivering a range of cargos into various cell types. The composite nature of these peptide arrangements allows the introduction of additional peptide‐mediated functionality, such as receptor binding peptides. This would allow the design of hybrid CPP/BBB functional complexes to strike the desired balance between CNS specificity, bidirectional BBB transport, and intraneuronal targeting.

## 4 CONCLUSION

Peptides represent an exciting class of compounds to readily functionalise cargos and improve BBB‐penetrating characteristics of a range of molecules. We highlight some of the most successful BBB and CPP peptides for delivery into the CNS. These are ready to be combined with diagnostic and therapeutic radiopharmaceuticals to develop highly effective CNS‐targeted agents.

## CONFLICT OF INTEREST STATEMENT

The authors declare no competing interests.

## Data Availability

Data sharing is not applicable to this article as no new data were created or analyzed in this study.
